# Worse clinical outcomes following percutaneous coronary intervention with a high SYNTAX score: A systematic review and meta-analysis

**DOI:** 10.1097/MD.0000000000007140

**Published:** 2017-06-16

**Authors:** Pravesh Kumar Bundhun, Akash Bhurtu, Feng Huang

**Affiliations:** a Institute of Cardiovascular Diseases, the First Affiliated Hospital of Guangxi Medical University; b Guangxi Medical University, Nanning, Guangxi, P.R. China.

**Keywords:** coronary artery bypass surgery, left main coronary artery diseases, multi-vessel coronary artery diseases, percutaneous coronary intervention, SYNTAX score

## Abstract

**Background:**

The synergy between percutaneous coronary intervention (PCI) with TAXUS and Cardiac Surgery (SYNTAX) score is an angiographic tool which is used to determine the complexity of coronary artery disease (CAD). We aimed to compare PCI versus coronary artery bypass surgery (CABG) in patients with a high SYNTAX score in order to confirm with evidence whether the former is really association with worse clinical outcomes.

**Methods:**

The National database of medical research articles (MEDLINE/PubMed), EMBASE database, and the Cochrane library were searched for publications comparing PCI versus CABG in patients with a high SYNTAX score, respectively. Death, myocardial infarction (MI), stroke, repeated revascularization, and a combined outcome death/stroke/MI were considered as the clinical endpoints. RevMan software was used to analyze the data, whereby odds ratios (OR) with 95% confidence intervals (CI) were used as the statistical parameters.

**Results:**

A total number of 1074 patients were included (455 patients with a high SYNTAX score were classified in the PCI group and 619 other patients with a high SYNTAX score were classified in the CABG group). A SYNTAX score cut-off value of ≥33 was considered relevant. Compared with CABG, mortality was significantly higher with a high SYNTAX score following PCI with OR: 1.79, 95% CI: 1.18 to 2.70; *P* = .006, *I*^2^ = 0%. The combined outcome death/stroke/MI was also significantly higher following PCI with a high SYNTAX score, with OR: 1.69, 95% CI: 1.24 to 2.30; *P* = .0009, *I*^2^ = 0%. In addition, PCI was also associated with significantly higher MI and repeated revascularization when compared with CABG, with OR: 3.72, 95% CI: 1.75 to 7.89; *P* = .0006, *I*^2^ = 0% and OR: 4.33, 95% CI: 1.71 to 10.94; *P* = .002, *I*^2^ = 77%, respectively. However, stroke was not significantly different.

**Conclusions:**

Compared with CABG, worse clinical outcomes were observed following PCI in patients with a high SYNTAX score, confirming with evidence, published clinical literatures. Therefore, CABG should be recommended to CAD patients who have been allotted a high SYNTAX score.

## 1 Introduction

Percutaneous coronary intervention (PCI) and coronary artery bypass surgery (CABG) are the 2 main revascularization procedures which are carried out in patients with left main or multi-vessel coronary artery disease (CAD).^[1]^ Newer scientific reports have already shown that CABG might be more beneficial and effective in patients with diabetes mellitus, complicated by multi-vessel CAD.^[2]^

The synergy between PCI with TAXUS and Cardiac Surgery (SYNTAX) score, an angiographic tool which is used to determine the complexity of CAD has shown to facilitate the selection of patients who might benefit from either PCI or CABG.^[3]^ It was derived from pre-existing classifications such as the American Heart Association (AHA) classification of CAD modified for the ARTS study, the American College of Cardiology (ACC)/AHA lesion classification, the Duke classification, the International classification for patient safety, and so on.

In patients who were allotted a low SYNTAX score, PCI was a good option whereas in patients who were allotted a high SYNTAX score, CABG was recommended.^[4]^ Even though this relevant tool has been used in clinical practice, very few research has provided evidence with data to support and further confirm this fact. Therefore, we aimed to compare PCI versus CABG in patients with a high SYNTAX score in order to confirm with evidence whether the former is truly association with worse clinical outcomes.

## 2 Methods

### 2.1 Data sources

Electronic databases: The National database of medical research articles (MEDLINE and its subgroup PubMed), EMBASE database, and the Cochrane library.

References: reference lists of relevant publications.

Official websites: official websites of most suitable journals of cardiology or cardiovascular diseases such as Circulation, the Journal of the American College of Cardiology, International Journal of Cardiology, and the American Journal of Cardiology were also searched for any relevant article.

### 2.2 Searched strategies

English publications were searched using the terms:

- “percutaneous coronary intervention and coronary artery bypass surgery and SYNTAX score”;
- “percutaneous coronary intervention and SYNTAX score”;
- “coronary artery bypass surgery and SYNTAX score”;
- “PCI, CABG, and SYNTAX score”;
- “SYNTAX score and revascularization”;
- “coronary artery disease and the SYNTAX score.”

### 2.3 Inclusion and exclusion criteria

Inclusion criteria were:

(a) Studies that consisted of patients with CAD.
(b) Studies comparing PCI versus CABG in patients with a high SYNTAX score, respectively.
(c) Studies that reported death, myocardial infarction (MI), stroke, major adverse cardiovascular and cerebrovascular events (MACCEs), or repeated revascularization as their clinical endpoints.

Exclusion criteria were:

(a) Studies that did not involve patients with CAD.
(b) Meta-analyses or letters to editors.
(c) Studies which did not compare PCI versus CABG in patients with a high SYNTAX score, respectively.
(d) Studies which did not report the above-mentioned clinical endpoints.
(e) Duplicated studies.

### 2.4 Types of participants

Patients with CAD were included in this analysis as shown in Table 1. However, when CAD was further subdivided, specific patients with:

(i) Left main coronary diseases;
(ii) Multi-vessel coronary diseases;
(iii) Three-vessel coronary diseases were included.

**Table 1:**
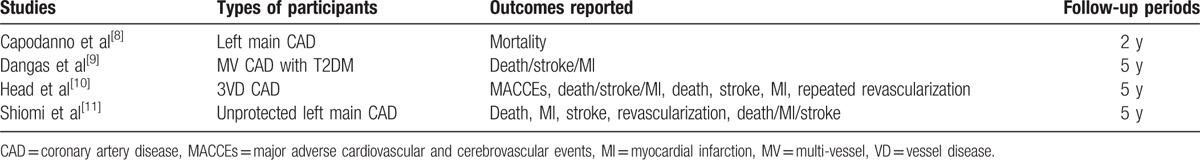
Types of participants, reported outcomes and follow-ups.

### 2.5 Outcomes and follow-ups

As shown in Table 1, the outcomes which have been analyzed in this study included:

(a) Mortality (all-cause death or cardiac death);
(b) Combined outcome including death/stroke/MI;
(c) MI;
(d) Stroke;
(e) Repeated revascularization.

MACCEs which were considered equally important, could not be analyzed since they were reported in only 1 study.

The follow-up periods varied from 2 years to 5 years as shown in Table 1.

### 2.6 Data extraction and quality assessment

This data extraction process was carried out by 2 independent reviewers (PKB and AB).

The following information was extracted:

Time of publication;

- Names of authors and names of trials or observational studies;
- Types of participants which were included;
- Reported outcomes and follow-up time periods;
- Methodological features of the trials;
- SYNTAX scores reported;
- Total number of patients which were classified in the PCI and CABG groups respectively;
- Baseline features of the patients.

The methodological quality of the trials was assessed with reference to the Cochrane collaboration^[5]^ and a score was allotted based on the presence of a low, moderate, or high bias risk. A minimum score of 0 (very high bias risk) and a maximum score of 12 (very low bias risk) were given.

Any disagreement which followed were discussed and solved by the third reviewer (FH).

### 2.7 Statistical analysis

This is a systematic review and meta-analysis of data reported in several previously published studies. Therefore, inconsistency across the studies was possible. However, 2 simple statistical tools were used to measure heterogeneity across the studies.^[6]^

(1) The *Q* statistic test, whereby a *P* value less or equal to .05 was considered statistically significant.
(2) The *I*^2^ statistic test. The higher the *I*^2^ value, the larger will be the heterogeneity. Therefore, a low *I*^2^ value could best represent a lower heterogeneity.

The statistical effects which were used were also dependent on the heterogeneity *I*^2^ value:

(1) If a low *I*^2^ value (<50%) was obtained for a specific group, a fixed effects model was used;
(2) If a high *I*^2^ value (>50%) was obtained when analyzing a particular subgroup, a random effects model was used.

The RevMan software version 5.3 was used to analyze the data, whereby odds ratios (OR) with 95% confidence intervals (CI) were the statistical parameters.

Patients’ consents and ethical or board review approval were not required.

## 3 Results

### 3.1 Searched outcomes

The PRISMA reporting guideline was followed.^[7]^

Electronic databases resulted in: 269 publications.

Primary exclusion based on titles and abstracts: 234 studies.

Full-text articles which were assessed: 35 studies.

Secondary exclusion:

- Meta-analysis (1)
- Did not compare PCI versus CABG in patients with a high SYNTAX score, but instead compared PCI or CABG separately with a low versus a high score respectively (8)
- Only compared PCI with a low SYNTAX score versus CABG with a high score (3)
- Duplicated studies (19)

**Figure 1:**
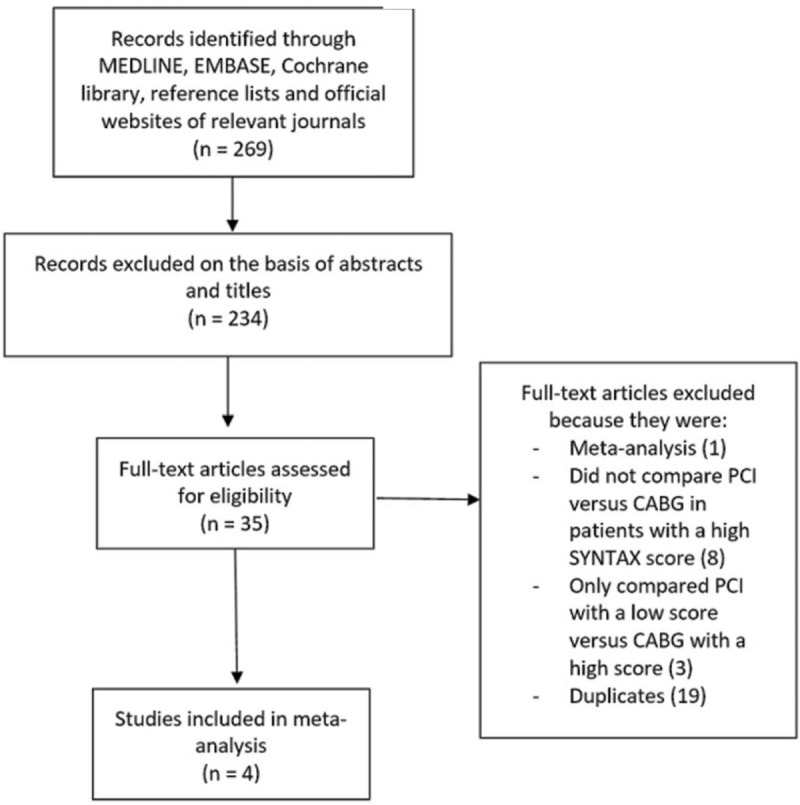
Flow diagram representing the study selection.

Finally, only 4 studies^[8–11]^ satisfied the inclusion and exclusion criteria and were selected for this analysis (Fig. 1).

### 3.2 General features of the studies which were included

As shown in Table 2, 2 studies were randomized controlled trials (SYNTAX and FREEDOM trials) and 2 studies were observational studies. A total number of 1074 patients were included (455 patients with a high SYNTAX score were classified in the PCI group and 619 patients with a high SYNTAX score were classified in the CABG group). A SYNTAX score cut-off value of ≥33 was considered relevant in this analysis. The exact number of patients with a high SYNTAX score was not provided in study Head2014, but the percentage of similar patients with adverse clinical outcomes was reported. Therefore, we assumed the total number of patients with a high SYNTAX score in that particular study to be 100 in each group as shown in Table 2.

**Table 2:**
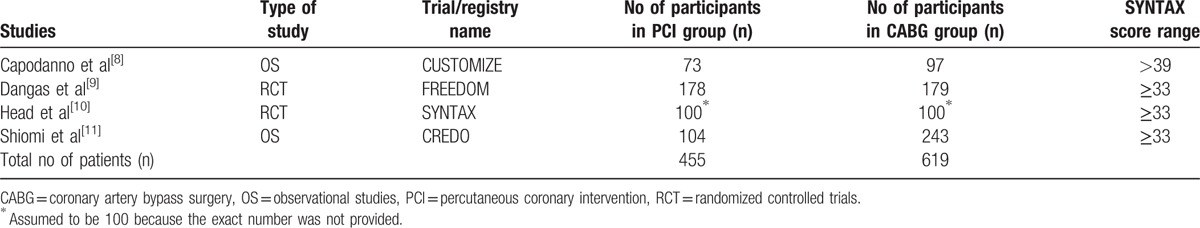
Main features of the studies which were included.

After a methodological assessment of the trials, a low risk of bias was observed in the SYNTAX and FREEDOM trials, and a score of 10 out of 12 points was allotted to each of them.

### 3.3 Baseline features of the patients

A mean age ranging from 62.6 to 76.8 years was reported among the patients. More than 60% of the patients (from the PCI and CABG groups) were male patients. Study Capodanno2011 reported 67.1% of man patients within the PCI group, and 69.1% of man patients within the CABG group. Study Head2014 reported 79.3% versus 81.1% of man patients within the PCI and CABG groups, respectively. All the 4 studies consisted of a majority number of patients with hypertension, with an increased percentage of more than 80 in 3 of the studies. Study Dangas2014 involved only patients with diabetes mellitus. Smoking history was highest in study Capodanno2011. Overall, when the groups (PCI versus CABG) were compared, there were no significant differences in baseline features among patients who were treated with PCI and CABG respectively as shown in Table 3.

**Table 3:**
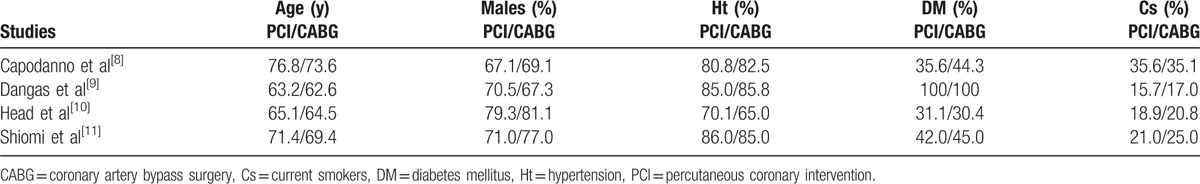
Baseline features of the studies which were included.

### 3.4 Main results of this analysis

**Figure 2:**
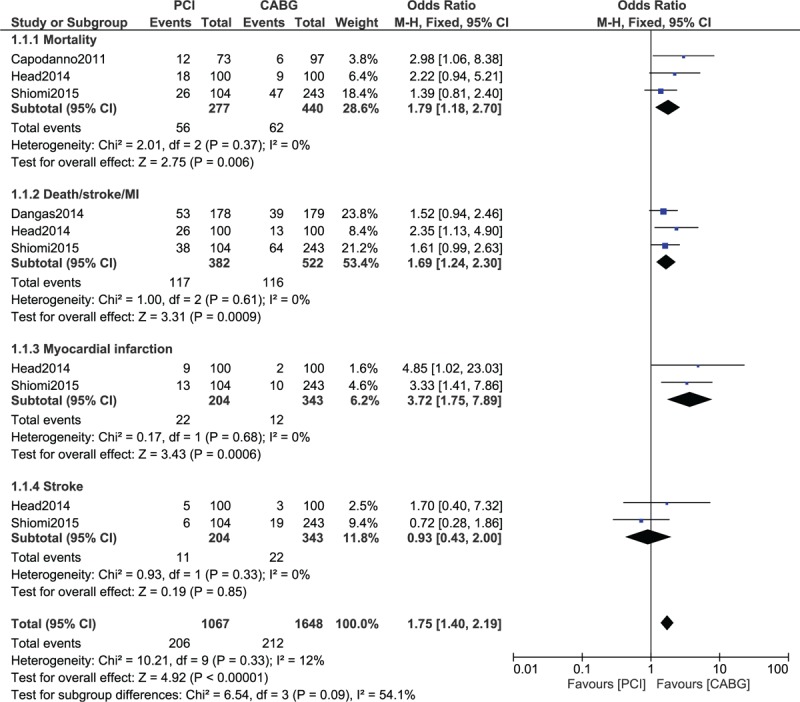
Adverse clinical outcomes observed in patients with a high SYNTAX score who were revascularized by CABG versus PCI. CABG = coronary artery bypass surgery, PCI = percutaneous coronary intervention.

**Figure 3:**
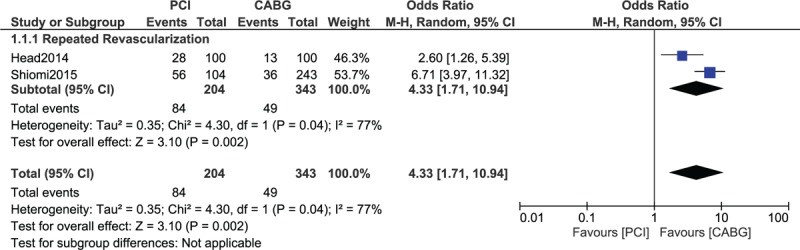
Repeated revascularization observed in patients with a high SYNTAX score who were treated by CABG versus PCI. CABG = coronary artery bypass surgery, PCI = percutaneous coronary intervention.

Results of this analysis (Table 4) showed that compared with CABG, mortality was significantly higher following PCI (high SYNTAX Score) with OR: 1.79, 95% CI: 1.18 to 2.70; *P* = .006, *I*^2^ = 0% (Fig. 2). The combined outcome death/stroke/MI was also significantly higher following PCI with a high SYNTAX score, with OR: 1.69, 95% CI: 1.24 to 2.30; *P* = .0009, *I*^2^ = 0% (Fig. 2). In addition, PCI was also associated with significantly higher MI (Fig. 2) and repeated revascularization (Fig. 3) in patients with a high SYNTAX score when compared with CABG, with OR: 3.72, 95% CI: 1.75 to 7.89; *P* = .0006, *I*^2^ = 0% and OR: 4.33, 95% CI: 1.71 to 10.94; *P* = .002, *I*^2^ = 77%, respectively. However, stroke was not significantly different with OR: 0.93, 95% CI: 0.43 to 2.00; *P* = .85, *I*^2^ = 0% (Fig. 2).

**Table 4:**
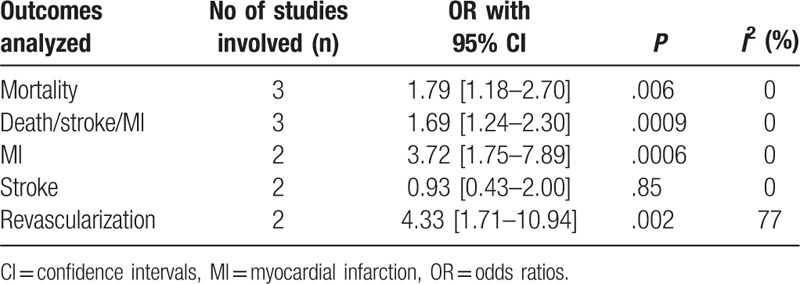
Results of this meta-analysis.

### 3.5 Publication bias

**Figure 4:**
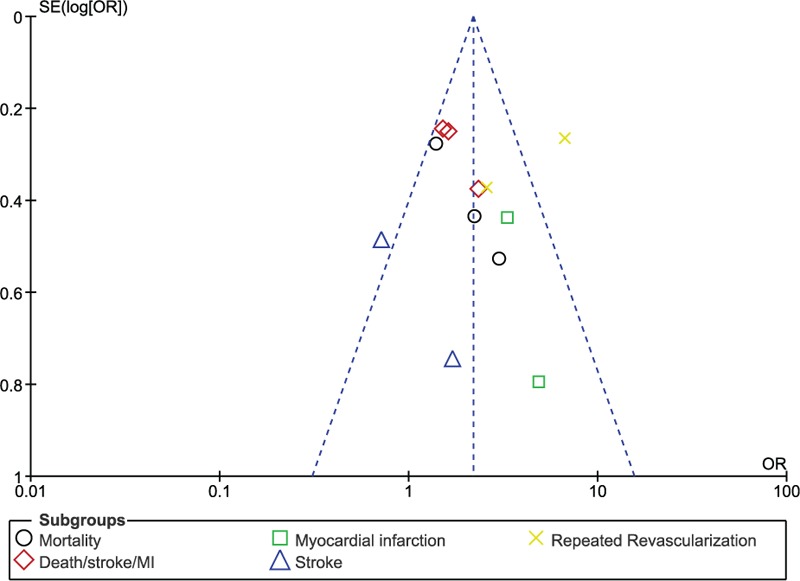
Funnel plot showing publication bias.

In this analysis, we selected studies which compared PCI versus CABG with a high SYNTAX score, respectively. Adverse clinical outcomes such as mortality, death/stroke/MI, repeated revascularization, stroke, and MI were assessed. Because this analysis involved only 4 studies, the only best way to assess publication bias was through funnel plots generated from RevMan 5.3. According to the funnel plot which was generated, a low evidence of publication bias was estimated across these 4 studies which assessed all the clinical endpoints as shown in Fig. 4.

## 4 Discussion

In this analysis, we aimed to compare PCI versus CABG in patients who were allotted a high SYNTAX score, respectively. Our results showed that PCI with a high SYNTAX score was associated with significantly higher mortality, combined death/stroke/MI outcome, MI, and repeated revascularization. However, stroke was not significantly different. This analysis has confirmed and has provided further evidence to the fact that CABG should benefit patients with a high SYNTAX score and that PCI should not be recommended to patients who have been allotted a high SYNTAX score, thus validating the SYNTAX score.

Another study validating the SYNTAX score also showed PCI in patients with a high SYNTAX score to be associated with worse clinical outcomes following this invasive procedure.^[12]^

The ARTS II registry also showed revascularization with PCI in patients with a higher SYNTAX score to result in worse clinical outcomes, therefore, showing the benefits of CABG in these patients.^[13]^ However, the cut-off values for the SYNTAX score were (16 and 24), whereas the cut-off value in this analysis was ≥33.

Similarly, a retrospective study showed that patients who were suggested CABG based on a high SYNTAX score, and who disagreed and preferred to be treated by PCI showed increased cardiac adverse outcomes.^[14]^ Even when the clinical SYNTAX score was used to predict treatment strategy, if patients with a high score disagreed to be revascularized by CABG and preferred to be treated by PCI, worse adverse outcomes were later observed.

Other applications of the SYNTAX score have been described.^[15–17]^ However, unfortunately the SYNTAX score has not shown to be useful in patients with non-ST segment elevation acute coronary syndrome who underwent CABG.^[18]^

Nevertheless, even if the use of the SYNTAX score has not significantly been generalized and popularized, another tool, which assesses a combination of the clinical and anatomical features of similar patients, the SYNTAX II score, is well-being appreciated and is showing effective predictive values.^[19]^ A recently published study further showed this SYNTAX II score to be even better in deciding the treatment strategy in patients with unprotected left main CAD, and reported a lower long-term mortality being associated with CABG compared with PCI with everolimus eluting stents (EES) in patients with higher SYNTAX II scores.^[20]^

### 4.1 Novelty

This research article is the first meta-analysis comparing PCI versus CABG in patients who were specifically qualified as having a high SYNTAX score. In addition, compared with previously published trials or observational studies, this analysis consisted of a larger number of patients. Another new feature could be the presence of a very low level of heterogeneity among several important subgroups assessing these clinical outcomes. The concept and idea of this research article are new, providing evidence, and analytical support to further confirm the existing literature concerning the SYNTAX score and its application. This analysis confirms the fact that patients allotted a high SYNTAX score should be revascularized by CABG which might be far more beneficial compared with PCI.

### 4.2 Limitations

Limitations could be the fact that a small sample size of patients was used during this analysis. Another limitation could be the fact that each subgroup analysis involved 2 or 3 studies. Moreover, patients with left main coronary disease and patients with multi-vessel coronary diseases were combined and analyzed. All the studies which were included had a follow-up period of 5 years except for 1 study, which had a follow-up period of only 2 years. Another limitation could be the fact that randomized patients and patients which were obtained from observational studies were combined and analyzed. Fortunately, a low level of heterogeneity was observed among several important subgroups.

## 5 Conclusions

Compared with CABG, worse clinical outcomes were observed following PCI in patients with a high SYNTAX score, confirming with evidence, published clinical literatures and validating the SYNTAX score. Therefore, CABG should be recommended to CAD patients who have been allotted a high SYNTAX score.
